# Mass production of bulk artificial nacre with excellent mechanical properties

**DOI:** 10.1038/s41467-017-00392-z

**Published:** 2017-08-18

**Authors:** Huai-Ling Gao, Si-Ming Chen, Li-Bo Mao, Zhao-Qiang Song, Hong-Bin Yao, Helmut Cölfen, Xi-Sheng Luo, Fu Zhang, Zhao Pan, Yu-Feng Meng, Yong Ni, Shu-Hong Yu

**Affiliations:** 1 Division of Nanomaterials and Chemistry, Hefei National Laboratory for Physical Sciences at the Microscale, Collaborative Innovation Center of Suzhou Nano Science and Technology, Department of Chemistry, CAS Center for Excellence in Nanoscience, Hefei Science Center of CAS, University of Science and Technology of China, Hefei, 230026 China; 2 CAS Key Laboratory of Mechanical Behavior and Design of Materials, Department of Modern Mechanics, University of Science and Technology of China, Hefei, Anhui 230027 China; 3 University of Konstanz, Physical Chemistry, Universitätsstraße 10, D-78457 Konstanz, Germany; 4 Advanced Propulsion Laboratory, Department of Modern Mechanics, University of Science and Technology of China, Hefei, 230026 China

## Abstract

Various methods have been exploited to replicate nacre features into artificial structural materials with impressive structural and mechanical similarity. However, it is still very challenging to produce nacre-mimetics in three-dimensional bulk form, especially for further scale-up. Herein, we demonstrate that large-sized, three-dimensional bulk artificial nacre with comprehensive mimicry of the hierarchical structures and the toughening mechanisms of natural nacre can be facilely fabricated via a bottom-up assembly process based on laminating pre-fabricated two-dimensional nacre-mimetic films. By optimizing the hierarchical architecture from molecular level to macroscopic level, the mechanical performance of the artificial nacre is superior to that of natural nacre and many engineering materials. This bottom-up strategy has no size restriction or fundamental barrier for further scale-up, and can be easily extended to other material systems, opening an avenue for mass production of high-performance bulk nacre-mimetic structural materials in an efficient and cost-effective way for practical applications.

## Abstract (web summary)

Artificial materials that replicate the mechanical properties of nacre represent important structural materials, but are difficult to produce in bulk. Here, the authors exploit the bottom-up assembly of 2D nacre-mimetic films to fabricate 3D bulk artificial nacre with an optimized architecture and excellent mechanical properties.

## Introduction

Natural structural materials generally exhibit exceptional mechanical performance relying on their complex hierarchical structures at multiple length scales^1–9^. Especially, nacre, as one of the most studied examples, composed of a highly ordered “brick-and-mortar” (BM) arrangement of brittle calcium carbonate platelets bonded by a thin layer of biopolymer, demonstrates how nature achieves these brilliant mechanical properties^10–12^. It has been motivating the fabrication of artificial nacre-inspired structural materials for a long time. High-performance nacre-inspired structural materials, especially these with large-sized three-dimensional (3D) bulk form, are attracting much more attention because of their promising applications for biomedicine, construction, aerospace, and military armor.

Traditional bottom-up assembly techniques, such as layer-by-layer strategies^13–15^, evaporation-induced self-assembly^16^, vacuum filtration^17^, and spray coating^18^, especially paper-making and doctor-blading^19^, have been intensively studied and are demonstrated to be environmental friendly, energy-efficient, economic, very efficient, and versatile for fabricating large-area, two-dimensional (2D) nacre-mimetic films with impressive nacre-like structures and mechanical properties^20, 21^. However, these techniques are restricted to the fabrication of 2D nacre-mimetic submillimeter-thick films, and it is difficult to produce 3D nacre-mimetic bulk materials. Other methods, such as uncontrolled co-casting^22^, extrusion and roll compaction^23^, as well as pressing and sintering of ceramic plates^24^, although possessing some advantages for fabricating 3D nacre-mimetic bulk materials, are limited by their insufficient microstructure control, and thus further mechanical enhancement of the prepared materials is still desirable. As promising alternatives, ice-templating and sintering of ceramics^25–28^, magnetic particle alignment^29, 30^, 3D printing^31^, and in situ growth by predesigned matrix-directed mineralization^32, 33^ were exploited recently, and some 3D bulk nacre-mimetic materials with good control over their hierarchical structures and mechanical performance have been successfully prepared. However, it is still a significant challenge for these techniques to be applied for further scale-up due to the complicated process, the high cost, and the low efficiency. Thus, more efficient approaches that allow mass production of 3D bulk nacre-mimetic structural materials are of great significance for further load-bearing applications.

Herein, we develop an efficient and versatile bottom-up approach to fabricate large-sized 3D bulk nacre-mimetic materials starting from individual inorganic microplatelets via combining well-developed evaporation-induced self-assembly with lamination technique^34, 35^. The proposed fabrication procedure takes advantages of nanoscale to micron-scale assembly induced by water evaporation and microscale to macroscale assembly by lamination, allowing the tuning of multiscale interface in the 3D bulk artificial nacre. Following this stepwise fabrication procedure, 3D nacre-mimetic bulks with half-meter width, several centimeters thickness, and superior mechanical performance can be facilely produced.

## Results

### Design and fabrication strategy

**Fig. 1:**
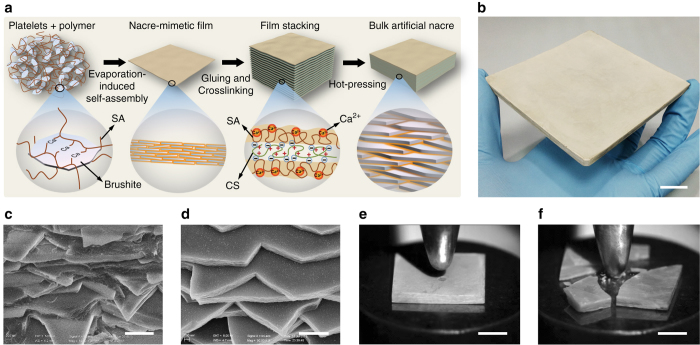
Fabrication and characterization of bulk artificial nacre. **a** Schematic illustration of the bottom-up assembly process of bulk artificial nacre. **b** Large as-fabricated bulk artificial nacre. *Scale bar*, 2 cm. **c**, **d** Cross-section of the artificial nacre **c** and natural *Cristaria plicata* nacre **d** show the similar fractured layered microstructure. *Scale bars*, 1 μm. **e**, **f** Artificial nacre **e** and *Cristaria plicata* nacre **f** under the same strength of impact, illustrating the higher impact resistance of the artificial nacre. *Scale bars*, 5 mm

The hierarchical structure and mechanical properties of the resulting artificial nacre were optimized at three different dimension levels from the molecular-level organization to the macroscopic structure according to previous theoretical guidance^10, 11, 15, 36^ (see Supplementary Discussion, Fig. 1a, and Supplementary Movie 1). In order to imitate the compositions of natural nacre, calcium phosphate (brushite) microplatelets (accessible in large scale) with similar sizes and mechanical properties to the aragonite platelets in natural nacre^10^ were used as the inorganic building blocks (Supplementary Fig. 1 and Supplementary Table 1). Biocompatible sodium alginate (SA) was used as the main biopolymer matrix because of its biocompatibility, flexibility of the polymeric chain, as well as the abundant carboxyl and hydroxyl groups on its molecular chain, which facilitate the interfacial interaction between the brushite platelets and SA through Ca^2+^–SA coordination^37^.

For the nanoscale to microscale assembly, large-area 2D nacre-mimetic films were prepared from the homogeneous mixture of brushite platelets and SA solution via water evaporation-induced self-assembly (Fig. 1a and Supplementary Movie 1), which can be easily scaled up by using a larger mould. The brushite–SA sol transformed to a hydrogel in a while after they were mixed (*left insert* in Supplementary Fig. 2a), suggesting a strong interfacial interaction between brushite platelets and SA molecules. The obtained films were translucent (Supplementary Fig. 2b, c), indicating the flat and uniform orientation of the brushite platelets in the polymer matrix^38^, which was further confirmed by scanning electron microscope (SEM) observation of the microstructure (Supplementary Fig. 3). At this stage, the mechanical properties of nacre-mimetic films were optimized by adjusting the size of brushite platelets and the mass ratio of the two constituents (Supplementary Figs. 4 and 5). The optimal brushite–SA composite film was determined to contain 50 wt.% of brushite platelets with an average size of ~8 μm and thickness of ~270 nm (see Supplementary Discussion).

To construct 3D bulk artificial nacre, the 2D nacre-mimetic films with equal size were then glued together with a thin layer of chitosan (CS) solution pre-sprayed on the surface of each film. The thin CS layer was expected to introduce strong electrostatic interaction at the interface between adjacent films via the coordination of carboxyl groups of SA and the amine groups of CS^39^ (Supplementary Fig. 6). After further pre-compression and drying, the bulk was subsequently soaked into CaCl_2_ solution to crosslink the SA molecules that interact with neither brushite platelets nor CS to enhance the strength of the organic matrix. After that, a hot-pressing step under higher pressure was applied to further improve the orientation degree of the platelets (Supplementary Fig. 7) and to increase the interfacial interactions in the bulk composites (Fig. 1a and Supplementary Movie 1). Through the above multistep facile process from a molecular to a macroscopic level, 3D bulk artificial nacre with large size (10 × 10 × 0.5 cm^3^) and low density (~1.8 g cm^−3^) in comparison with natural *Cristaria plicata* nacre and previously reported bulk nacre-mimics were obtained^26, 27, 30, 40^ (Fig. 1b and Supplementary Table 2). SEM observations of the microstructure of the final bulk artificial nacre revealed a distinct step-like layered structure at the fracture surface, which was strikingly similar to that of natural *C. plicata* nacre (Fig. 1c, d and Supplementary Fig. 8). Furthermore, this bottom-up approach can be extended to other material systems such as clay-based and mica-based composites, resulting in large and thick nacre-mimetic bulk materials (16 × 16 × 2.5 cm^3^ for clay and 50 × 50 × 1 cm^3^ for mica, which can be further increased easily; Supplementary Fig. 9).

### Investigation of mechanical properties

**Fig. 2:**
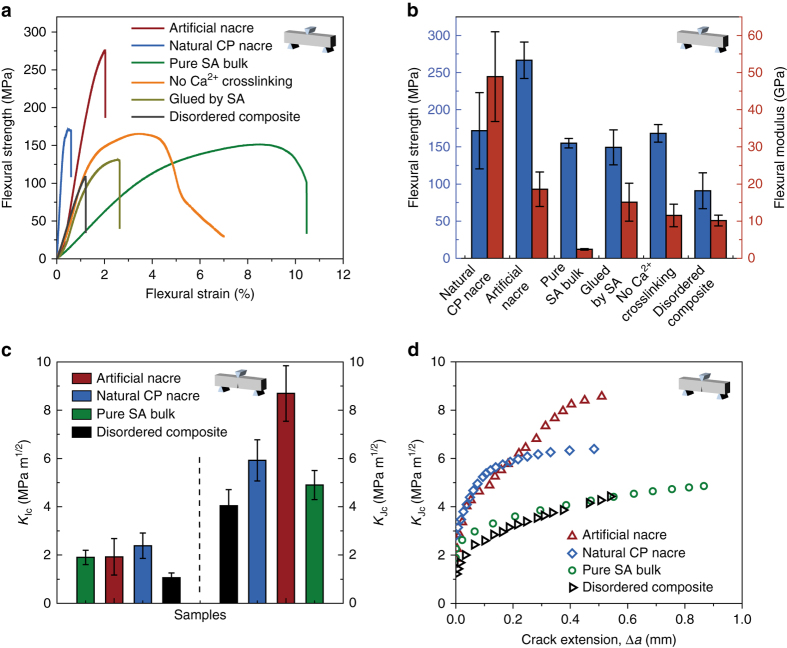
Mechanical properties of as-fabricated bulk artificial nacre. **a** Flexural stress–strain curves of the artificial nacre fabricated with different synthetic conditions, natural *Cristaria plicata* (CP) nacre, pure SA bulk, and disordered composite. **b** Comparison of flexural strength and stiffness of the artificial nacre fabricated with different synthetic conditions with natural CP nacre, pure SA bulk, and disordered composite. **c** Fracture toughness for crack initiation (*K*_Ic_) and stable crack propagation (*K*_Jc_) of the bulk artificial nacre compared to natural CP nacre, pure SA bulk, and disordered composite. **d** Crack-resistance curves (R-curves) showing the resistance to fracture in terms of the stress intensity, *K*_Jc_, as a function of crack extension, Δ*a*, for the bulk artificial nacre, natural CP nacre, pure SA bulk, and disordered composite. All the *error bars* represent the s.d. of at least six replicate measurements

The mechanical properties of the final bulk artificial nacre were then systematically studied and compared to that of the natural *C. plicata* nacre, pure SA bulk, and disordered brushite–SA composite in order to demonstrate the validity of our multiscale nacre-mimetic structural design. As shown in Fig. 1e, f and Supplementary Movie 2, under the same strength of shock, artificial nacre maintains its integrity while natural *C. plicata* nacre is cracked into smaller pieces. The impact strength of the artificial nacre was measured as ~7.1 KJ m^−2^, which is slightly lower than that of pure SA bulk (~10.5 KJ m^−2^), but five times larger than that of natural *C. plicata* nacre (~1.4 KJ m^−2^). It means that the designed artificial nacre is a little brittler than pure SA bulk, while tougher than natural *C. plicata* nacre. Furthermore, a significant increase for both the strength and stiffness of the artificial nacre was observed after crosslinking by Ca^2+^ (Fig. 2a, b and Supplementary Table 3), which can be attributed to the mechanical enhancement of the organic matrix arising from the Ca^2+^–SA coordination at the molecular level (see also the Supplementary Discussion). At the microscopic level, it was found that the incorporation of brushite platelets with high orientation degree greatly contributed to the mechanical performance of the final artificial nacre. As revealed in Fig. 2a, b and Supplementary Table 4, simply incorporating brushite platelets into the SA matrix without structural control can only improve the stiffness of the composite, but it seriously compromises other mechanical properties, while the ultimate flexural strength and stiffness of the bulk artificial nacre with “BM” microstructure are both much higher than those of pure SA bulk and the disordered brushite–SA composite made by simply mixing the constituents. At the macroscopic level, the ultimate flexural strength and the stiffness decreased when the laminated brushite–SA films were glued together by SA solution instead of CS solution (Fig. 2a, b and Supplementary Table 3), which further confirmed the significant contribution of the interfacial electrostatic interaction between the positively charged CS and negatively charged SA molecules.

In addition, the mechanical properties of the final bulk materials are also influenced by the thickness of the secondary building films and the applied pressure in the hot-pressing process (Supplementary Fig. 10 and Supplementary Table 3). The optimized thickness of films in our system was determined to be ~75 μm. Higher pressure led to higher ultimate flexural strength of as-fabricated artificial nacre, which could be ascribed to the denser arrangement and higher orientation degree of the inorganic platelets under higher applied pressure. The ultimate flexural strength of the nacre-mimic bulk can be up to ~267 MPa after the hot-pressing process of 100 MPa, further surpassing that of natural *C. plicata* nacre (~172 MPa). However, the ultimate stiffness (~18.6 GPa) of the nacre-mimic bulk is still less than that of natural *C. plicata* nacre (~48.9 GPa) because of the high organic content (66.4% vol. for the nacre-mimic bulk calculated from mass fraction, in contrast to 5% vol. for natural nacre^10^), which might be greatly improved when extending this bottom-up strategy to pure ceramic material systems^27^. Moreover, consistent with the 2D individual films, the 3D bulk artificial nacre exhibiting the best mechanical performance is also composed of ~50 wt.% of brushite platelets (Supplementary Fig. 10f and Supplementary Table 5).

The fracture toughness, *K*_Ic_, as an evaluation of the resistance to crack initiation, was measured to be a little lower for the bulk artificial nacre (~1.9 MPa m^1/2^) than that for natural *C. plicata* nacre (~2.4 MPa m^1/2^), while higher than that of the disordered brushite–SA composite (~1.2 MPa m^1/2^) and equal to pure SA bulk (~1.9 MPa m^1/2^), partly confirming the effective role of the designed nacre-mimetic multiscale microstructure. The J–R-curve approach, which has been used in similar artificial structural materials and a wide range of natural materials^26, 27, 41–43^, was applied here to describe the multiple extrinsic toughening mechanisms of the bulk artificial nacre. The results reveal that the bulk artificial nacre exhibits similar extensive rising R-curve behavior as that of natural *C. plicata* nacre (Fig. 2d), indicating their resistance to fracture during crack propagation. The maximum fracture toughness, *K*_Jc_, of the artificial nacre increases by more than three times from the crack initiation (~1.9 MPa m^1/2^) to the end of the stable crack propagation (~8.7 MPa m^1/2^), which surpasses that of natural *C. plicata* nacre (~5.9 MPa m^1/2^) and pure SA bulk (~5.9 MPa m^1/2^), and far exceeds that of the disordered brushite–SA composite (~3.8 MPa m^1/2^). These results strongly illustrate that the bulk artificial nacre possesses both high strength and toughness similar to natural structural materials, which can be attributed to the multiscale replication of the hierarchical “BM” structure of natural nacre.

### Toughening mechanisms analysis

**Fig. 3:**
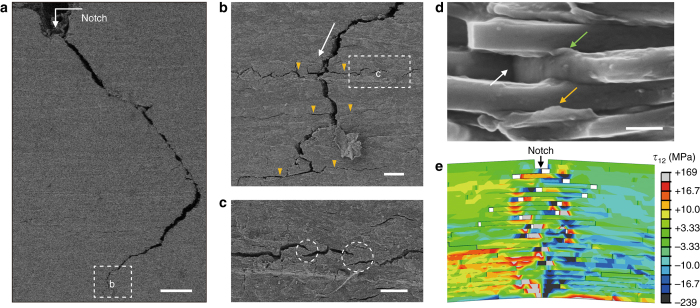
Multiple extrinsic toughening mechanisms acting at multiple length scales. **a** Long-range crack deflection. *White arrow* indicates the initial crack near the notch. *Scale bar*, 200 μm. **b** Crack deflection, branching, and multiple cracking. *White arrow* indicates the extension direction of the main crack. *Yellow arrows* indicate the onset of crack branching. *Scale bar*, 20 μm. **c** Crack bridging toward the end of a crack path. *Scale bar*, 10 μm. **d** Details of the fracture surfaces showing polymer bridging, stretching, and platelets’ pulling out. *White arrow* indicates the cavity resulted from platelet pull-out. *Green arrow* shows the polymer bridging between adjacent platelets. *Yellow arrow* displays that the polymer stretching resulted from interface failure. *Scale bar*, 400 nm. **e** Microcrack deflection and crack bridging near the crack tip by progressive interface failure via nonlinear finite element model (FEM) simulation. *τ*_12_ means the shear stress

The multiple extrinsic toughening mechanisms acting at several length scales were further investigated via the fracture mechanics analysis and corresponding theoretical simulation. In situ observation of the specimens under single-edge notched bending tests displays that the morphology of the extended cracks in the bulk artificial nacre and pure SA bulk is obviously different (Supplementary Fig. 11 and Supplementary Movie 3). Figure 3a and Supplementary Fig. 11a show that the crack in the bulk artificial nacre initiates from the notch and propagates along a tortuous path. This typical crack deflection phenomenon followed by significant interface failure is identified as one of the most dominant extrinsic toughening mechanisms for the high fracture resistance of natural materials and other bioinspired structural materials^27, 42–44^. In contrast, the crack straightly extends opposite to the notch in the pure SA specimen (Supplementary Fig. 11b and Supplementary Movie 3). This result provides another good evidence for the contribution of our multiscale designed nacre-mimetic microstructure to the toughness enhancement of the bulk artificial nacre, which agrees well with the experimental data in Supplementary Table 4. Higher-magnification SEM images of the fracture surface further illustrate the presence of crack branching (Fig. 3b), multiple cracking (Fig. 3b), and crack bridging at the crack tip (Fig. 3c), which are all identified as effective extrinsic toughening mechanisms that act to decrease the crack-driving force in natural structural materials^8, 42–44^. Moreover, SEM observation of the fracture surface indicates extensive interface delamination between brushite platelets, and characteristic trapezoidal structure is revealed (Supplementary Fig. 8a, b), with great similarity to the fracture surface of natural *C. plicata* nacre (Supplementary Fig. 8c, d). Notably, a polymer layer that densely adhered on platelets’ surfaces can be observed at the fracture surface (Fig. 3d), implying strong platelet–SA interfacial interaction. Furthermore, distinct polymer bridging, stretching due to interface failure, and cavities generated by platelets’ pull-out are found (Fig. 3d), which is believed to lead to the efficient energy dissipation by frictional sliding and breaking of polymer matrix when the crack encountered the platelet–polymer interface^45^. All the proposed extrinsic toughening mechanisms synergistically redistribute the applied load and effectively relieve the locally high stresses at different length scales, thus resulting in the rising R-curve behavior of the bulk artificial nacre (Fig. 2d). In all, the extrinsic toughening derived from the “BM” hierarchical architecture played a crucial role on the load redistribution and toughness enhancement in bulk artificial nacre.

Moreover, nonlinear finite element modeling simulation (see the details in Supplementary Discussion and Supplementary Fig. 12) shows distinct microcrack deflection near the crack tip by progressive interface failure in a typical “BM” structure (Fig. 3e and Supplementary Movie 5). The further interface failure dominated by interface sliding with friction, plasticity, and platelets’ pull-out, accompanied with daughter microcrack nucleation and branching, is identified as crack bridging at a larger scale (Fig. 3e, Supplementary Fig. 13 and Supplementary Movie 4). These simulated structural features under crack propagation are consistent with those we observed in the bulk artificial nacre (Fig. 3a–d). The toughening behavior predicted in the crack bridging model displays a similar rising crack-resistance curve as shown in the experimental results (Fig 2d and Supplementary Fig. 14), which further indicates the successful replication of mechanical behaviors of natural nacre into the bulk artificial nacre through the multiple-scale structural mimicking.

## Discussion

**Fig. 4:**
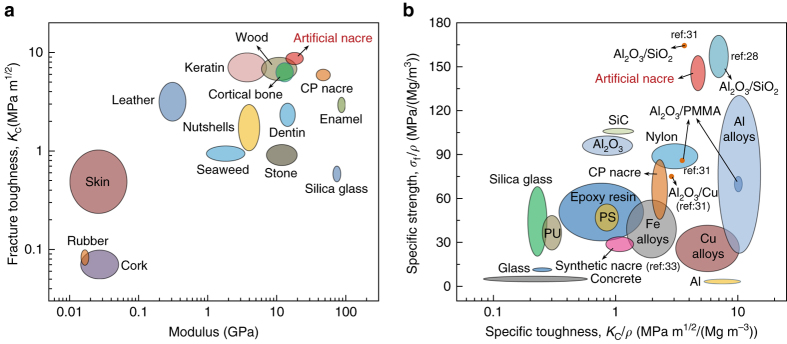
Comparison of mechanical properties of our artificial nacre with its competitors. **a** Ashby diagram of the fracture toughness of our bulk nacre-mimics compared with a wide range of natural structural materials as a function of their stiffness^49, 50^. **b** Ashby diagram of specific strength vs. specific toughness for our bulk nacre-mimics compared with a range of synthetic engineering materials^8, 26, 27, 30, 32, 49^

Similar to natural nacre, the hierarchical “BM” structure in the bulk artificial nacre induces the combination of both enhanced strength and toughness without obvious compromise. Figure 4a shows that the fracture toughness and flexural modulus of the bulk artificial nacre are both higher than those of a wide range of natural materials such as bone, enamel, dentin, and nacre. Meanwhile, the artificial nacre proves to be a typical light-weight, strong, and tough material. As shown in Fig. 4b, both the specific strength (σ_f_/*ρ*) and specific toughness (*K*_f_/*ρ*) of the bulk artificial nacre are higher than those of various engineering materials including high-performance ceramics and metallic alloys. The mechanical superiority of the fabricated bulk artificial nacre endows it with great potential for future structural applications, especially for bone substitute materials in biomedicine due to its similar compositions. Since other platelets, like clays, and functional nanoplatelets, like vanadia, can be assembled by similar approaches, our reported strategy shows a broad potential also for a variety of other materials.

Following such a designed two-level, bottom-up assembly approach, a 3D bulk artificial nacre with centimeter size was facilely achieved. Based on the theoretical guidance, the hierarchical structure from molecular level to macroscopic level was optimized, and both the structural and mechanical features of natural nacre were successfully replicated in the bulk artificial nacre. The obtained bulk artificial nacre exhibits impressive strength, fracture toughness, and impact resistance, which confirms the role of artificial hierarchical “BM” structure. Although the combination of light-weight and high strength, toughness, and impact resistance may not be superior to that of some existing engineering materials using nacre-like microstructures, further improvement of the mechanical performance could be expected if we extend the fabrication method into other potential composite system with the stronger building blocks. Furthermore, this method is highly feasible and efficient, and can be further scaled up easily. This work, in principle, highlights a practical guideline for further development of more high-performance, bioinspired structural materials on the basis of combinatorial bottom-up assembly strategies.

## Methods

### Preparation of brushite (CaHPO_4_·2H_2_O) platelets

Brushite platelets with an average lateral size of ~8 µm and thickness of ~270 nm were prepared via a modified earlier work^46^. In a typical process, 1.650 g of KH_2_PO_4_ and 15.192 g of Na_2_HPO_4_·12H_2_O were dissolved in 1.4 L deionized water (DIW). An amount of 6.062 g of CaCl_2_ was dissolved in 0.4 L DIW. These two solutions were placed in a 2 ^o^C water bath for 30 min. Then, the CaCl_2_ solution was poured into the other solution, followed by stirring for ~90 min. Finally, the white precipitates were collected by filtration and dried in a 60 ^o^C oven for 24 h. For brushite platelets with average lateral sizes of ~18 and ~30 µm and thicknesses of ~500 nm and~680 nm, the reaction temperatures were kept at 12 and 25 ^o^C, respectively.

### Fabrication of brushite–SA nacre-mimetic films

SA powder was dissolved into DIW to obtain 2 wt.% SA solution, and then centrifuged (8000 rpm, 10 min) to remove the undissolved impurities. Appropriate amounts of brushite platelets and SA solution were mixed together by vigorously stirring for ~20 min, followed by ultrasonic treatment for 30 min, and then was treated by vacuum-pumping to remove the air bubbles. After that, the mixture was carefully poured into polytetrafluoroethylene molds and placed in ambient condition for water evaporation-induced self-assembly. After ~36 h freestanding dried nacre-mimetic films were obtained.

### Fabrication of bulk materials

CS powder was dissolved into acetic acid solution to obtain 1 wt.% CS solution, and then centrifuged (8000 rpm, 10 min) to remove the undissolved impurities. For ordered bulk artificial nacre, the large nacre-mimic films were cut into pieces with equal sizes and spray-coated with a thin layer of CS solution on the surface. They were then laminated together by stacking and were pre-pressed with 5 MPa at 60 ^o^C for 24 h. The bulk hybrid composite was immersed into CaCl_2_ solution (1 mol L^−1^) for 2 h and rinsed three times with DIW. After that, a second hot-pressing step with certain pressure (25, 50, or 100 MPa) was applied at 80 ^o^C for ~24 h. For pure SA bulk, 5 wt.% SA solution was carefully poured into polytetrafluoroethylene molds and placed in ambient condition for water evaporation. After the solution changed to gel, the gel was cut into equal-sized pieces, which were then laminated together after being wetted with DIW. When the gel stack was dried in ambient condition, it was immersed into CaCl_2_ solution (1 mol L^−1^) for 2 h and then dried in a 60 ^o^C oven after being rinsed with DIW. For disordered bulk composite, the mixture of brushite and SA solution was freeze-dried and mashed into powder. The powder was then spray-coated with the equal amount of CS solution with that for preparation of ordered bulk artificial nacre. After the above steps, the mixture experienced the same hot-pressing procedure to fabricate the disordered hybrid composite.

### Sample characterizations

X-ray diffraction of patterns was carried out on a PW1710 instrument with CuKα radiation (*λ* = 0.15406 nm). Fourier transform infrared spectroscopy (FTIR) spectra were obtained from a Bruker Vector-22 FTIR spectrometer at room temperature. The transmittance of the nacre-mimetic films was measured on UV-2501PC/2550 at room temperature (Shimadzu Corporation, Japan). Element content in the bulk artificial nacre was quantitatively analyzed by elemental analyzer (Vario EL cube, Elementar). The microstructure of the all the samples was observed by SEM (Zeiss Supra 40) at an acceleration voltage of 5 kV. The evolutions of the bulk artificial nacre and natural nacre under strong shock were captured by a high-speed video camera (FASTCAM SA5, Photron Limited). The dynamic crack extensions in the bulk artificial nacre and pure SA bulk under a three-point bending test were captured by digital microscope (B011, Shenzhen D&F co., LTD, China). Density of the bulk artificial nacre and natural nacre was determined from their total mass divided by their total volume.

### Mechanical testing

The mechanical property of the brushite platelets was measured by using Nano Indenter G200 system (Agilent Technologies). Tensile tests of nacre-mimetic films and three-point bending tests of bulk artificial nacre were carried out on Instron 5565 A using 500 N load cells. Impact strength of the artificial nacre was measured using Charpy Impact Tester (XJJY-5, Chengde Bao Hui Testing Machine Manufacturing co., LTD, China). The specimens of natural nacre were cut from freshwater mussel (*C. plicata*). All the mechanical tests were conducted at room temperature with relative humidity of ~50%. At least six specimens were tested for all the values presented. For heavy hammer impact test, the hammer with 500 g weight was falling freely from 20 cm height, and the tested specimens were 2 mm thick for both the artificial nacre and natural *C. plicata* nacre. For tensile mode testing, the film samples were cut into ~2 mm wide and ~15 mm long stripes. Tests were performed at a loading rate of 1 mm min^−1^ with a gauge length of 8 mm. For three-point bend tests, the specimens were carefully cut with a thickness *B* ~2 mm and width *W* ~2 mm. Tests were performed at a loading rate of 0.2 mm min^−1^ with a support span of 12.5 mm. For single-edge notched bend (SENB) tests, the samples (with a thickness *B* ~2 mm and width *W* ~2 mm) were first notched to ~50% of its width by using a 150 µm-thick diamond raw and then sharpened by slightly sliding a razor blade repeatedly; the final notch radius is about 50 µm. Tests were performed at a constant displacement rate of 1 µm s^−1^. For beam impact test, the specimens were cut into specimens with the length of ~80 mm, width of ~10 mm, and thickness of ~4 mm. Vickers hardness measurements were carried out using microhardness tester (HX-1000, Suzhou AOKA Optical Instruments co., LTD) with an applied maximum load of 1.0 kg for 10 s. For both Vickers hardness measurements and three-point bending tests, the applied loading direction was perpendicular to micro-platelet basal surface.

### Mechanical calculation

Fracture toughness, *K*_Ic_, was calculated using following equations^40^.

1 $${K_{{\rm{Ic}}}} = \frac{{{P_{{\rm{Ic}}}}S}}{{B{W^{3/2}}}}f\left( {a/W} \right)$$

2 $$f\left( {a/W} \right) = \frac{{3{{\left( {a/W} \right)}^{1/2}}\left[ {1.99 - \left( {a/W} \right)\left( {1 - a/W} \right)\left( {2.15 - 3.93a/W + {{\left( {a/W} \right)}^2}} \right)} \right]}}{{2\left( {1 + 2a/W} \right){{\left( {1 - a/W} \right)}^{3/2}}}}$$

In which, *P*_Ic_ is the maximum load in SENB test, *S* is support span, *B* is the thickness of the specimen, *W* is the width of the specimen, and *a* is the notch depth.

Fracture toughness, *K*_Jc_, in this work was calculated from the elastic and plastic contribution, which relates to J-integral calculation, similar to previously reported method for measuring the properties of bone^42, 47^ and other artificial composites^27, 28, 31, 48^.

3 $$J = {J_{{\rm{el}}}} + {J_{{\rm{pl}}}}$$

*J*_el_ is the elastic contribution, which is based on linear elastic fracture mechanics.

4 $${J_{{\rm{el}}}} = \frac{{{K_{{\rm{Ic}}}}^2}}{{E'}}$$

The plastic contribution *J*_pl_ can be calculated with the following equation,

5 $${J_{{\rm{pl}}}} = \frac{{2{A_{{\rm{pl}}}}}}{{B\left( {W - a} \right)}}$$

*A*_pl_ is the plastic area underneath the load-displacement curve.

Thus, *J* values can be transformed into *K* values by the following equation:

6 $${K_{{\rm{Jc}}}} = {\left( {JE'} \right)^{1/2}}$$

Where, $E' = E(1 - {v^2})$, *E* is Young’s modulus, and *v* is the Poisson’s ratio. As the variation of *E* influence *K*_Jc_ in a fairly limited way; here, *E*′ can be replaced by *E*.

Crack extension, Δ*a*, was calculated according to previously reported indirect method^28, 31^ with the following equations:

7 $${a_n} = {a_{n - 1}} + \frac{{W - {a_{n - 1}}}}{2}\frac{{{C_n} - {C_{n - 1}}}}{{{C_n}}}$$

8 $${C_n} = {u_n}{\rm{/}}{f_n}$$

9 $$\Delta a = {a_n} - a$$

where *a*_*n*_ and *C*_*n*_ are the crack length and complaisance calculated at each point after the departure of the creak, respectively. *u*_*n*_ and *f*_*n*_ are the displacement and force at each point after departure of the crack, respectively. *W* is the width of the specimen.

### Finite element analysis of the BM structure

A 3D nonlinear finite element model is developed using the commercial software ABAQUS v6.13. In the simulation, a 3D BM structure (250 × 60 × 2 µm^3^) with a single-edge notch (2 µm × 4 µm × 2 µm) is adopted, as shown in Supplementary Fig. 12a. The BM structure in the FE model contains a randomly staggered arrangement of bricks bonded by the thin layer of biopolymer, which is modeled as a cohesive zone with a bilinear traction separation and undergoes dry friction after damage. The bricks with isotropic bulk modulus *E*_p_ = 100 GPa, Poison ratio *v*_p_ = 0.33, and the failure strength $\sigma _{\rm p}^m$ = 200 MPa bear elastic deformation before brittle failure. The bilinear constitutive response of traction separation law is shown in Supplementary Fig. 12b. The initial response of the cohesive element is assumed to be linear until a damage initiation criterion is met. The energy-based Benzeggagh and Kenane (BK) damage evolution criterion with a mixed mode fracture is adopted. In the standard BK option in Abaqus, when the accumulated energy release rates *G* (*G* = *G*_I_+*G*_II_) is larger than the critical energy release rate *G*_c_, the interface is fully fractured. Here *G*_c_ is defined as ${G_{{\rm{Ic}}}} + \left( {{G_{{\rm{IIc}}}} - {G_{{\rm{Ic}}}}} \right){\left( {\frac{{{G_{{\rm{II}}}}}}{{{G_{\rm{I}}} + {G_{{\rm{II}}}}}}} \right)^{\!\!\eta} }$ with the BK material parameter *η* being 1.45, the critical energy release rate *G*_Ic_=3N m^−1^, and *G*_IIc_=5 N m^−1^ for mode I and mode II, respectively. We simulated the crack propagation with the three-point bending. The ends of top/bottom are fixed and the loading are applied at the center of the bottom/top. We have chosen the parameters in the Abaqus model such that the flexural stress-displacement curve in our numerical three-point bending test shown in Supplementary Fig. 12c can recover to the similar experimental three-point bending test shown in Fig. 2a (see the details in Supplementary Discussion).

### Data availability

The data that support the findings of this study are available on request from the corresponding authors (S.-H.Y. or Y.N.).

## Electronic supplementary material

Supplementary Information

Supplementary Movie 1

Supplementary Movie 2

Supplementary Movie 3

Supplementary Movie 4
