# Supplementary material for: Mass production of bulk artificial nacre with excellent mechanical properties
Source: Nat Commun. 2017 Aug 18;8:287. doi: 10.1038/s41467-017-00392-z (PMC5562756; doi:10.1038/s41467-017-00392-z)
Supplement: Supplementary file 1 — Supplementary Information [file 41467_2017_392_MOESM1_ESM.pdf]

Description of Supplementary Files

File Name: Supplementary Information
Description: Supplementary Figures, Supplementary Tables, Supplementary Discussion and Supplementary References

File Name: Supplementary Movie 1
Description: Schematic representation shows the fabrication process of the 3D bulk artificial nacre.

File Name: Supplementary Movie 2
Description: High speed camera video shows the process of natural and artificial nacre under heavy hammer impact.

File Name: Supplementary Movie 3
Description: In situ observation of the crack extension of the 3D bulk artificial nacre and pure SA bulk using under single-edge notched bending tests.

File Name: Supplementary Movie 4
Description: Microcrack deflection and crack bridging near the crack tip by progressive interface failure via nonlinear finite element model simulation.

Supplementary Discussion

Volume-fraction and size dependent strength

The microstructure dependent strength of the nacre-mimetic composite can be understood using the mechanics of composite structure[1-6]. According to a modified rule of mixture formula[1, 2], the tensile strength of the composite has the form $\sigma_c = ø\sigma_p^m + (1\text{-}ø)\,\sigma_{int}^m$ with $ø$ the volume fraction of the platelet, σ_p^m and σ_{int}^m the tensile strength of the platelet and interlayer, respectively. Usually σ_{int}^m is much smaller than σ_p^m, and thus can be neglected. The value of σ_p^m depends on the failure mode of the nacreous composite[3-5]. Using the shear lag model the maximum tensile stress in the platelets is estimated as $\tau^f l_p / h_p$ with τ^f the shear strength of the interlayer, and l_p / h_p the aspect ratio of the platelet, if the value of $\tau^f l_p / h_p$ is larger than the Griffith strength $(\gamma_p E_p / h_p)^{1/2}$ with γ_p, E_p the surface energy and Young's modulus of the platelet, respectively, the platelet fracture is favorable and $\sigma_p^m \approx (\gamma_p E_p / h_p)^{1/2}$, otherwise $\sigma_p^m \approx \tau^f l_p / h_p$ in the case of interface failure dominated.

The derived estimation of σ_c can explain why the increase of the interface adhesion by Ca^{2+} coordinated ionic bonds and the decrease of the platelet thickness (h_p) effectively enhances the strength of the composite shown in Fig. 2b and Supplementary Fig. 4. In addition, it also explains that σ_c increases with the increase of $ø$. However, our experimental result further reports that a non-monotonic volume fraction dependency. The σ_c has a maximum when the $ø$ is close to a critical value (Supplementary Figs. 5 and 10e). We believe the reason is possibly due to the fact that the further increase of $ø = h_p / \left(h_p + h_{int}\right)$ (with h_p, h_{int} the thicknesses of the platelets and the interlayer matrix, respectively) has to increase h_p, or decrease h_{int}. As our experimental result shown

in Supplementary Fig. 4, the increase of $ø$ causes the decrease of h_{int}, which then leads to inhomogeneous distribution of the polymer between the platelets (Supplementary Fig. 4f), thus reduces the effective τ^f of the interlayer and the strength of the composite $\sigma_c \approx ø\tau^f l_p / h_p$ in the case of interface failure dominated.

Theoretic analysis of toughening mechanisms

The impressive feature of the nacre-mimetic composite is its capability to mitigate the strength-ductility tradeoff by arresting the crack at the interface followed by controllable interface failure. There are two kinds of interface dominated toughening mechanisms confirmed in our nacre-mimetic materials.

One is the microcrack deflection at the interface between platelets. If the geometry of the platelet is in the optimal range, i.e. the thickness of the platelet is tens of to a few hundred nanometers and the aspect ratio of the platelet is close to the ratio of the ultimate strengths between the platelet and interlayer[6], the cracks in the platelets fail to grow and the interface failure dominates. In addition, the staggering randomness to a certain extent further alters the shear stress at the interfaces and leads to a progressive interface failure and a highly meandering microcrack path[3]. In our experiments, the thickness of platelets is several hundred nanometers, and their aspect ratio is 20-50 in consistent with the theoretical prediction[5], such parameter selection guarantees large amount of interface failure sufficiently to dissipate the elastic energy. The observed multiple crack bifurcation and daughter crack nucleation in Fig. 3 may be the proof of highly meandering microcrack due to interface failure.

The other toughening mechanism is crack bridging at a larger scale. It is known that with the increase of the applied loading, the further interface failure is dominated by interface sliding with friction, plasticity and platelets pulling out. Near the crack tip, the platelets bridge the crack and form a

crack-bridging zone, as sketched in Supplementary Fig. 14a. By using a simple crack-bridging model[7], the increase of the stress intensity due to the crack bridging has the form $\Delta K = 2\left(2/\pi\right)^{1/2}\sigma_0\Delta a^{1/2}$ with σ_0 the cohesive strength in the Dugdale's cohesive law and Δa the size of the crack bridging zone which is roughly proportional to the crack extension. As shown in Supplementary Fig. 10c, the applied stress intensity factor $K_{Jc} = K_{Ic} + \Delta K$ with K_{Ic} characterizing the intrinsic toughness will monotonically increase with the increase of Δa before the crack-bridging zone is saturated ($\Delta a \le \lambda_{br}^{SS}$ with λ_{br}^{SS} the steady state length of the crack-bridging zone), in a good agreement with the *R*-curve in Fig. 2d, where K_{Jc} can be much larger than K_{Ic} indicating significant extrinsic toughening. Obviously, large crack-bridging zone is the key factor of extrinsic toughening. A rigorous fracture model is proposed to discuss how the development of the crack-bridging zone depends on the size, the volume fraction and arrangements of the platelets as well as the mechanical properties of the platelets and the interlayer[7]. The result in the model further provides guideline for the rational design of tough nacreous composites.

Finite element analysis of the brick-and-mortar structure.

A three-dimensional (3D) nonlinear finite element model is developed using the commercial software ABAQUS v6.13. In the simulation, a 3D brick-and-mortar (BM) structure (250 × 60 × 2 μm^3) with a single edge notch (2 × 4 × 2 μm^3) is adopted, as shown in Supplementary Fig. 1a. The BM structure in the FE model contains a randomly staggered arrangement of bricks bonded by the thin layer of bio-polymer which is modeled as a cohesive zone with a bilinear traction-separation and undergoes dry friction after damage. The bricks with isotropic bulk modulus E_p=100 GPa, Poisson ratio v_p = 0.33 and the failure strength σ_p^m = 200 MPa bear elastic deformation before brittle failure. In

bilinear constitutive response of traction-separation law shown in Supplementary Fig. 1b, T represents the interfacial strength, δ_c is critical separation, δ_f is the separation at failure and the area under the curve, G_c, is the critical strain energy release rate. In 3-point bending simulation, a mixed mode fracture is under consideration. The properties of mix mode fracture are required to define two critical fracture energies $G_{Ic} = 3\ \text{N}\cdot\text{m}^{-1}$ and $G_{IIc} = 5\ \text{N}\cdot\text{m}^{-1}$ in bilinear traction-separation law, wherein the modulus related to penalty stiffness is $K_1 = 1\ \text{GPa}$ for tensile deformation and $K_2 = 0.8\ \text{GPa}$ for shear deformation, and the interfacial strengths $T = 30\ \text{MPa}$ for tensile deformation and $S = 45\ \text{MPa}$ for shear deformation. The characterized thickness of cohesive element is $t_c = 1\ \mu\text{m}$. The initial response of the cohesive element is assumed to be linear until a damage initiation criterion is met. The penalty stiffness, K_i of the bi-linear traction-separation law is defined as

$$K_i = \frac{T_i}{\delta_{ci}} \tag{1}$$

where i=1 and 2. And i=1 stands for tensile deformation and i=2 stands for shear deformation. The definition of i is also applied for following equations. We choose the quadratic stress as the damage initiation criterion:

$$\left(\frac{\langle\sigma\rangle}{T}\right)^2 + \left(\frac{\tau}{S}\right)^2 = 1 \tag{2}$$

where σ is tensile stress, τ is shear stress, and the Macaulay bracket, $\langle\sigma\rangle$ represents that the compressive stress does not contribute to the damage initiation. Once the damage initiation, the stiffness begins to degrade. The softening response of cohesive element of cohesive element is

$$\sigma_i = (1-d) K_i \Delta_i \tag{3}$$

where d is scalar stiffness degradation (SDEG in Abaqus), which has the value d = 0 when the interface is undamaged, and the value d = 1 when the interface is fully fractured. The energy-based Benzeggagh and Kenane (BK) damage evolution criterion is adopted[8]

$$G_c = G_{Ic} + \left(G_{IIc} - G_{Ic}\right)\left(\frac{G_{II}}{G_I + G_{II}}\right)^{\eta} \tag{4}$$

with the BK material parameter η being 1.45, the critical energy release rate for accumulated of mode I and mode II, G_{Ic} and G_{IIc}. In the standard BK option in Abaqus, as the accumulated energy release rates G ($G = G_I + G_{II}$) is larger than the critical energy release rate G_c, the interface is fully fractured. Here, G_I and G_{II} is the calculated energy release rate for mode I and mode II, respectively. Friction coefficient between adjacent bricks is 0.2. We simulated the crack propagation with the three-point bending. The ends of top/bottom are fixed and the loading are applied at the center of the bottom/top. We have chosen the parameters in the Abaqus model such that the flexural stress-displacement curve in our numerical three-point bending test shown in Supplementary Fig. 1c can recover to the similar experimental three-point bending test shown in Fig. 2a.

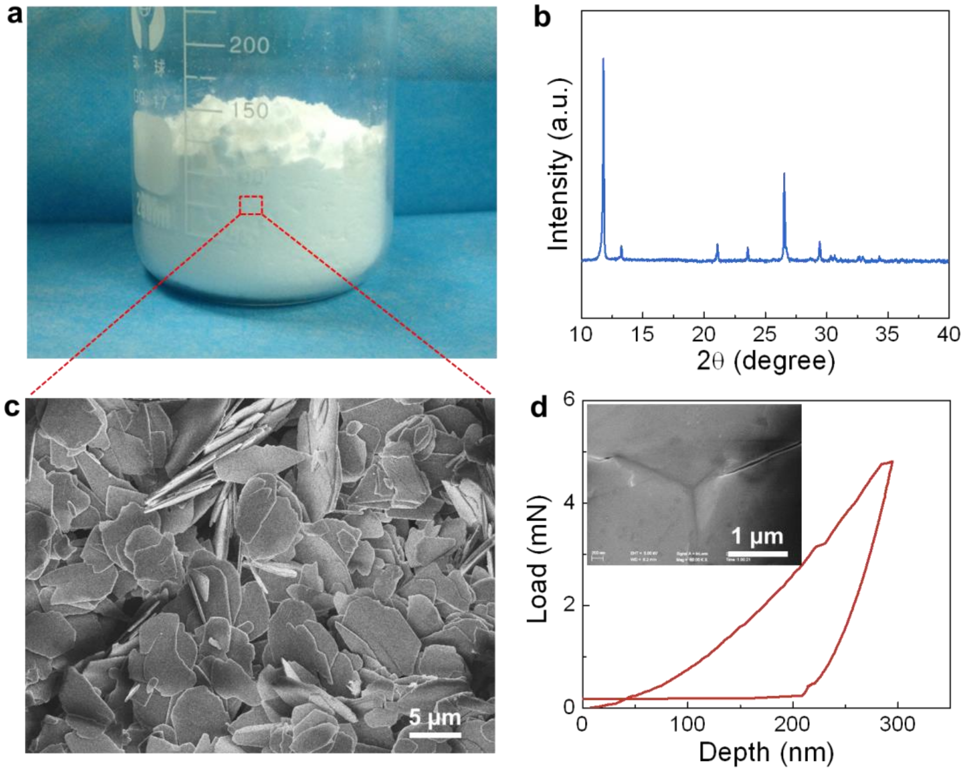

Supplementary Figure 1 | Characterization of brushite platelets. a, Digital photograph shows the synthesized brushite platelets powders at large scale. **b**, X-Ray Diffraction (XRD) pattern of brushite platelets. **c**, SEM image shows the micro-sized artificial brushite platelets. **d,** Stress-strain curve of a nanoindentation test of a brushite platelet (with average lateral size of about 30 μm, thickness of about 680 nm). Insert SEM image shows the indentation on the surface of the brushite platelet.

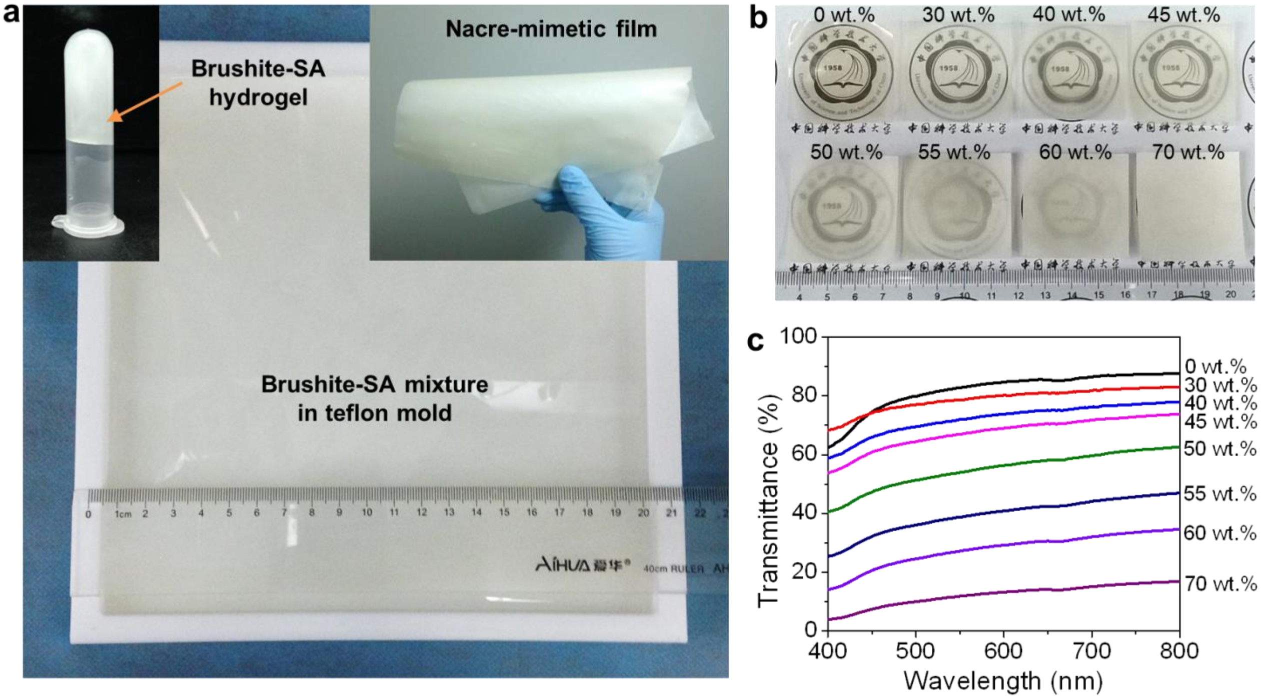

Supplementary Figure 2 | Fabrication and characterization of 2D nacre-mimetic films. a, The digital photograph shows the brushite-SA mixture contained in a mold for the preparation of large-area nacre-mimetic films by water evaporation induced self-assembly. The insert indicates that the brushite-SA sol transformed to a hydrogel in a while after they were mixed, suggesting a strong interfacial interaction induced by the coordination between Ca^{2+} at the surface of brushite platelets and the SA molecules. **b**, The digital photograph displays the as-prepared large-area nacre-mimetic film. The size of the nacre-mimetic film is determined by the size of the mould and the thickness depends on both the volume and the concentration of the mixtures in the mould. **c**, A series of nacre-mimetic films with different degree of transparence composed of different content of inorganic brushite platelets.

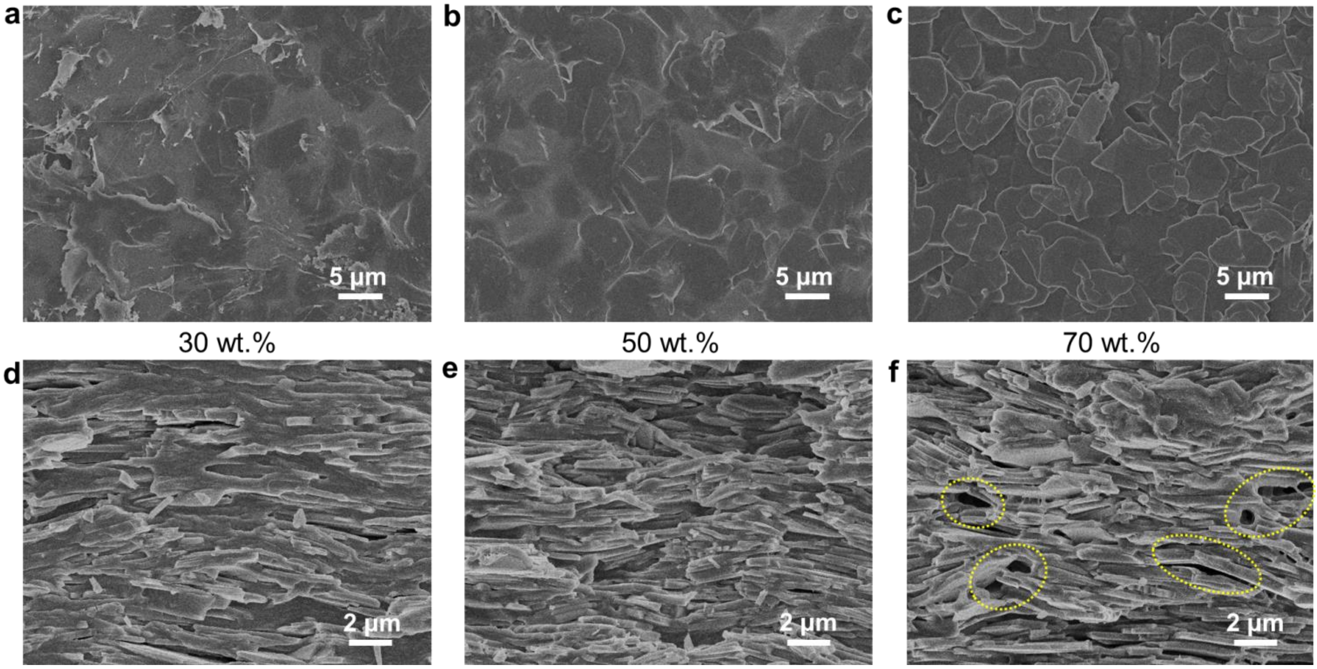

Supplementary Figure 3│Microstructure of the as-fabricated nacre-mimetic films. a-**c**, SEM images show the surface of the nacre-mimetic films with different platelets contents. **d**-**f**, Cross-sectional SEM images show the layered brick-and-mortar structure of the nacre-mimetic film with different platelets contents. As the platelets content increases some cavities among the platelets appear (marked in yellow circles).

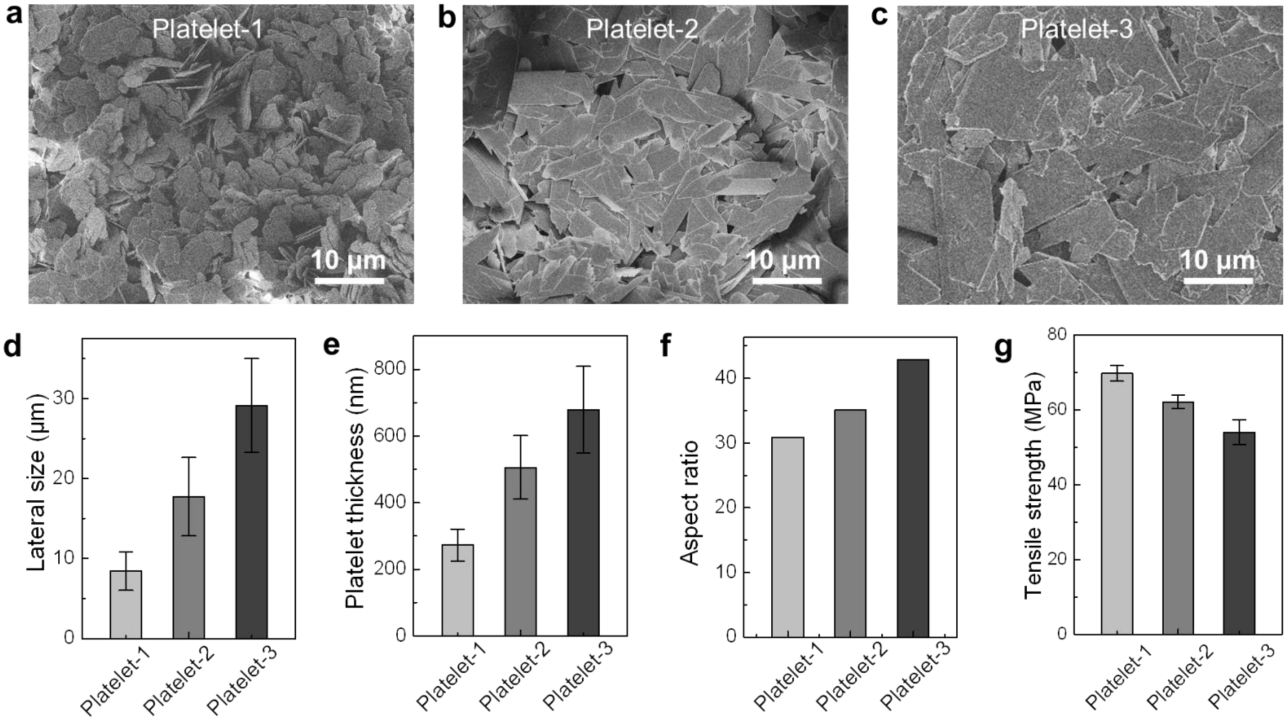

Supplementary Figure 4│Strength of the nacre-mimetic films assembled from brushite platelets with different size. a-c, SEM images show the synthetic brushite platelets with different sizes. **d-f**, Plotted graphs show the average diameter, thickness and aspect ratio of the three kinds of brushite platelets, which were determined by calculating them from SEM images, where at least 20 measurements of the brushite platelets were taken. The thickness of the platelets was measured from the cross-sectional SEM image of nacre-mimetic films assembled from the relevant platelets. **g**, Tensile strength of the nacre-mimic films assembled from these platelets (50 wt%). All the error bars represent the s.d. of at least six replicate measurements.

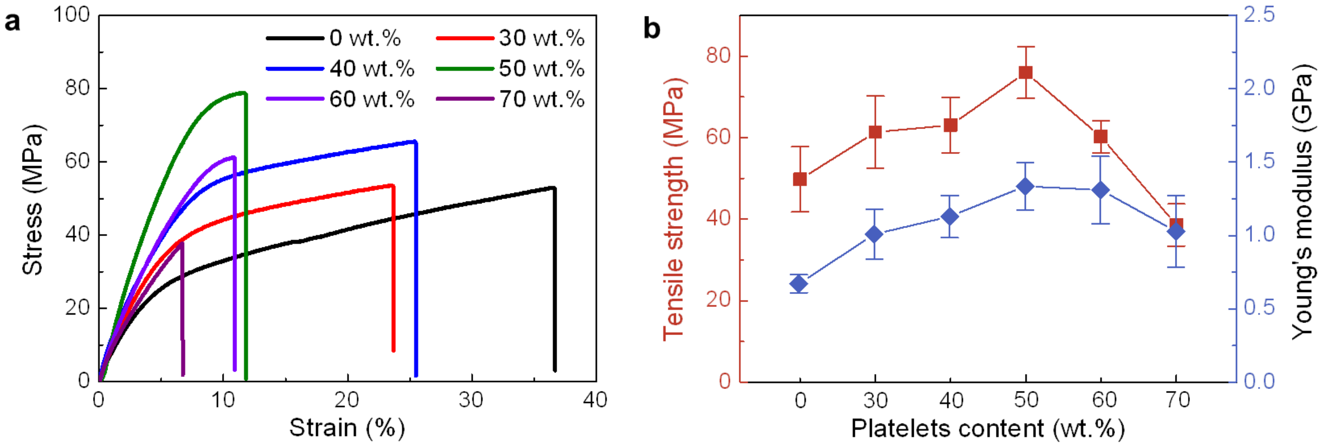

Supplementary Figure 5 | Mechanical properties of the nacre-mimetic films containing different contents of brushite platelets. **a**, Stress-strain curves of the nacre-mimic films consists of different content of inorganic platelets. **b**, Comparison of the tensile strength and modulus of the nacre-mimic films consists of different content of inorganic platelets. All the error bars represent the s.d. of at least six replicate measurements.

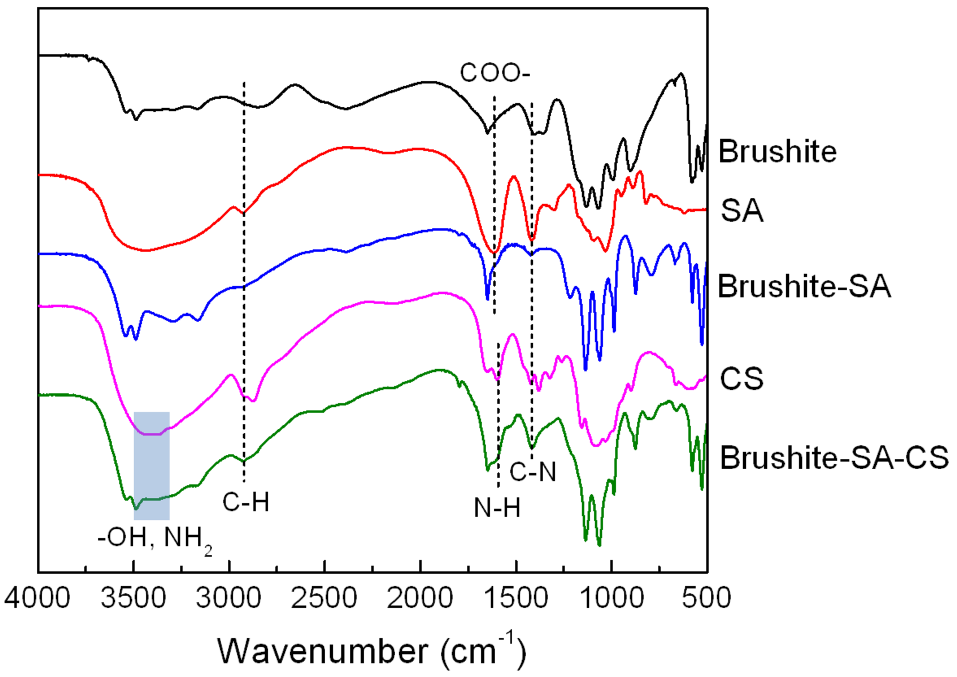

Supplementary Figure 6│Fourier transform infrared spectroscopy (FTIR) characterization. FTIR spectra of brushite platelets, SA, brushite-SA (it means SA coated brushite platelets, which were obtained by mixing brushite platelets and SA solution and subsequently washing by DIW for three times), CS, and brushite-SA-CS (it means CS coated brushite-SA, which was obtained by mixing brushite-SA and CS solution and subsequently washing by DIW for three times). The band at 2926.0 cm^{-1} is due to C-H antisymmetric stretching vibration. The band at 1615.3 cm^{-1} is attributed to COO- antisymmetric stretching vibration. The FTIR spectrum for CS showed the distinctive absorption bands at 3350-3500 cm^{-1}, indicating the combination of stretching vibration of the -OH and $-NH_2$. The absorption at 1556 cm^{-1} is designated as N-H. The emerging of the C-H and COO- bands in brushite-SA after brushite platelets coated with SA indicates that SA molecules are adsorbed on the surface of brushite platelets due to the Ca^{2+} coordinated ionic bonds between SA and the platelets. The enhancement of C-H band and the emerging of the of N-H band in brushite-SA-CS after brushite-SA coated with CS indicates that CS molecules are adsorbed on the surface of brushite-SA due to the electrostatic adsorption between SA and CS.

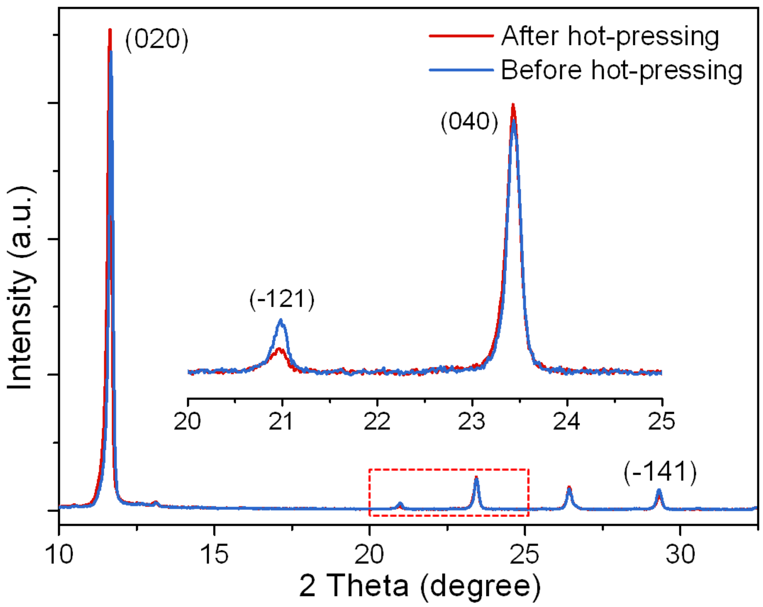

Supplementary Figure 7 | X-Ray Diffraction (XRD) patterns of the nacre-mimetic bulk before and after hot-pressing. The orientation degree of brushite platelets in the nacre-mimetic bulk before and after hot-pressing process was measured according to previously described method[9]. In which, the degree of preferred orientation is calculated from

$$\eta = 100\%[\frac{(1-r)^3}{1-r^3}]^{\frac{1}{2}}, \tag{5}$$

where the March parameter r is given by

$$r = [\frac{\sin^2\alpha}{(\frac{\kappa}{\kappa_p})^{\frac{2}{3}} - \cos^2\alpha}]^{\frac{1}{3}}. \tag{6}$$

In the above equation, α is the angle between two crystal planes, κ is the intensity ratio of the two crystal faces, and κ_p is the value of κ for a random powder.

X-ray diffraction (XRD) measurements were first carried out to measure the intensity ratio κ. The XRD patterns (Fig. 4) show a very strong preferred orientation of the [020] type for the nacre-mimetic bulk before and after hot-pressing process and other diffraction lines are strongly suppressed. Two representative peaks (020) and (-121) were selected. The angle α between them is 0.97.

The XRD data of brushite R070554 (RUFF database: http://rruff.info/) was used to calculate κ_p.

$$\kappa_p = \frac{I(020)}{I(-121)} = 0.06525. \tag{7}$$

Based on the measured XRD data of the nacre-mimetic bulk, we have

$$\kappa_{\text{Before hot-pressing}} = \frac{I(020)}{I(-121)} = 71.51, \tag{8}$$

$$\kappa_{\text{After hot-pressing}} = \frac{I(020)}{I(-121)} = 111.11. \tag{9}$$

Thus, based on Supplementary equation (6), we get

$$r_{\text{Before hot-pressing}} = [\frac{\sin^2\alpha}{(\frac{\kappa_{\text{Before hot-pressing}}}{\kappa_p})^{\frac{2}{3}} - \cos^2\alpha}]^{\frac{1}{3}} = 0.185823, \quad (10)$$

$$r_{\text{After hot-pressing}} = [\frac{\sin^2\alpha}{(\frac{\kappa_{\text{After hot-pressing}}}{\kappa_p})^{\frac{2}{3}} - \cos^2\alpha}]^{\frac{1}{3}} = 0.168445. \quad (11)$$

Consequently, based on Supplementary equation (5), the orientation degrees of brushite platelets in the nacre-mimetic bulk before and after hot-pressing process are

$$\eta_{\text{Before hot-pressing}} = 100\%[\frac{(1-r_{\text{Before hot-pressing}})^3}{1-r_{\text{Before hot-pressing}}^{\ 3}}]^{\frac{1}{2}} = 73.7\%, \quad (12)$$

$$\eta_{\text{After hot-pressing}} = 100\%[\frac{(1-r_{\text{After hot-pressing}})^3}{1-r_{\text{After hot-pressing}}^{\ 3}}]^{\frac{1}{2}} = 76.0\%. \quad (13)$$

Thus, we can draw a conclusion that the orientation degree of the platelets in the bulk composites is improved in the hot-pressing step.

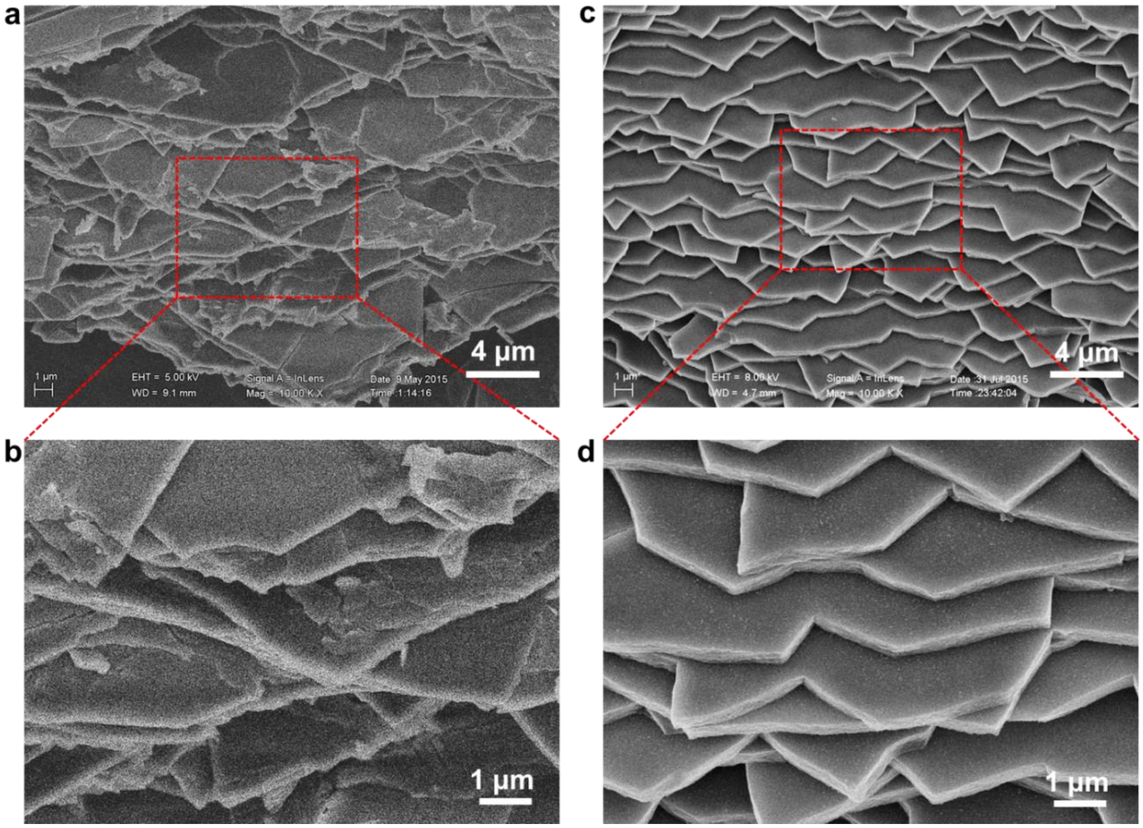

Supplementary Figure 8│Microstruture observation. **a**, **b**, SEM images show the fracture surface of the bulk artificial nacre. **c**, **d**, SEM images show the fracture surface of natural *Cristaria plicata* nacre.

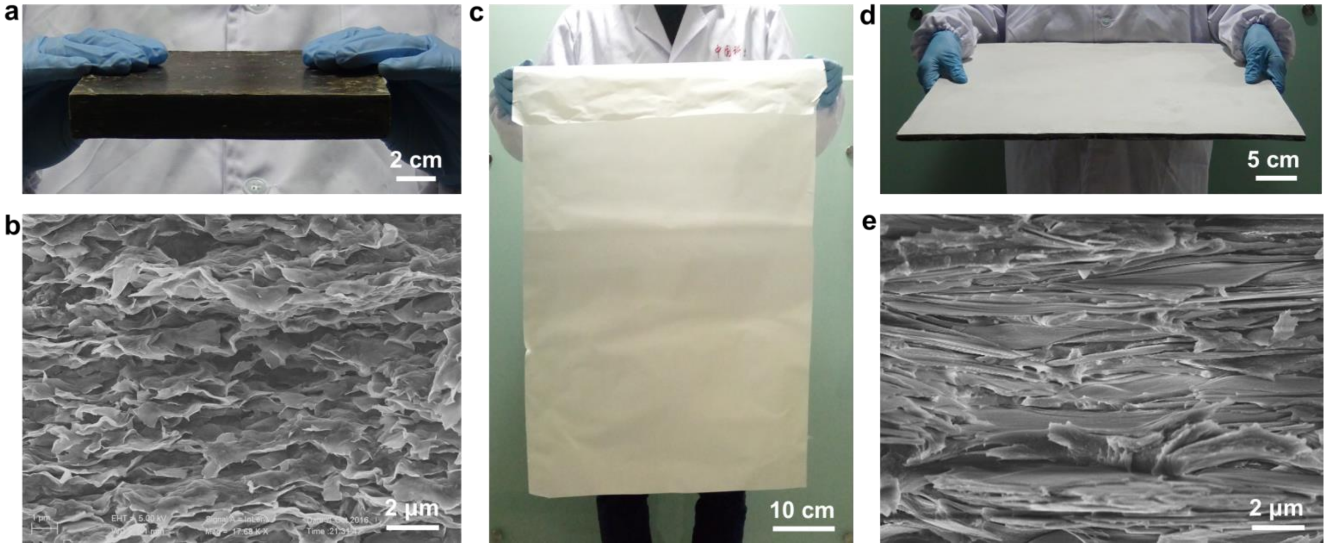

Supplementary Figure 9│Large-sized 3D bulk nacre-mimetic materials. a, b, Photograph (**a**) and sectional view SEM image (**b**) of 3D bulk nacre-mimetic material assembled from montmorillonite nano-platelets and SA with CS as inter-film glue. **c,** Photograph of large-sized 2D nacre-mimetic film assembled from mica micro-platelets and SA. **d, e,** Photograph (**d**) and sectional view SEM image (**e**) of 3D bulk nacre-mimetic material assembled from mica micro-platelets and SA with CS as inter-film glue.

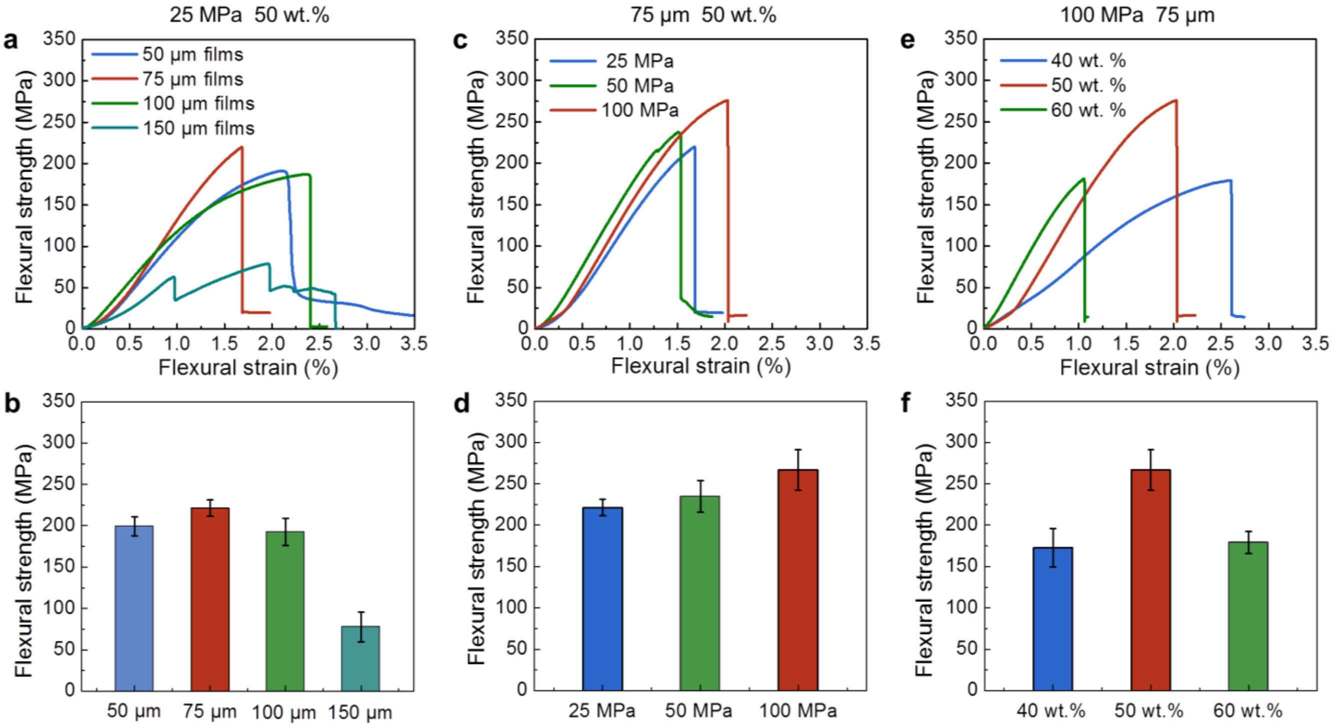

Supplementary Figure 10 │ Comparison of flexural strength of the artificial nacre prepared at different conditions. a, **b**, Flexural stress-strain curves and plotted diagram of the artificial nacre made from nacre-mimetic films with different thickness. In this case, the applied pressure is 25 MPa and platelet content is 50 wt.%. **c**, **d**, Flexural stress-strain curves and plotted diagram of the artificial nacre fabricated with different pressures in the hot-pressing process. In this case, platelet content is 50 wt.% and nacre-mimetic films with ~75 μm were selected. **e**, **f**, Flexural stress-strain curves and plotted diagram of the artificial nacre with different contents of brushite platelets. In this case, the applied pressure is 100 MPa and nacre-mimetic films with ~75 μm were selected. The flexural strength of the obtained artificial nacre decreased with the increasing thickness of the films and the delamination occurred in the bending tests when the film thickness increased to ~150 μm. It can be explained that the number of the interfaces between adjacent films decreased when thicker films were laminated into a bulk material with equal thickness. Thus, the sliding-resistant contribution from the interfacial electrostatic interactions became less, resulting in weaker mechanical strength of the final bulk solids. On the other hand, if the applied films were too thin, it would be hard to handle these films, which might lead to some defects in the as-fabricated bulk artificial nacre and deteriorate its mechanical properties. All the error bars represent the s.d. of at least six replicate measurements.

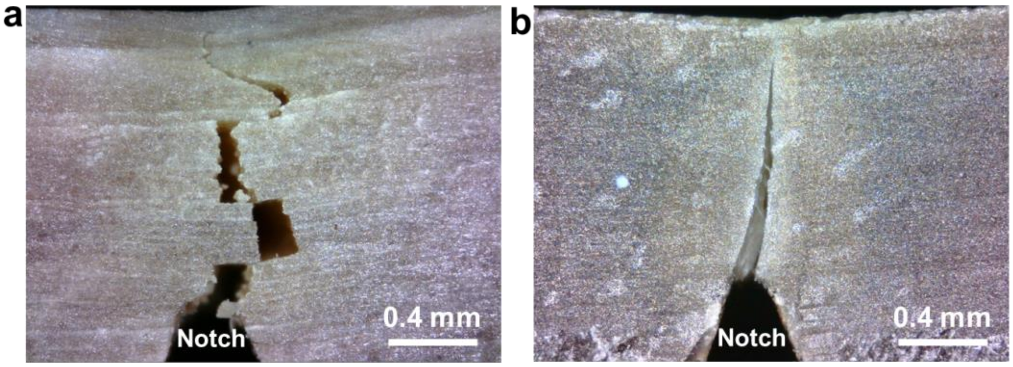

Supplementary Figure 11 | *In situ* observation of crack extension under optical microscope. Crack propagation in the designed bulk artificial nacre (**a**) and pure SA bulk (**b**) under single-edge notched bending tests.

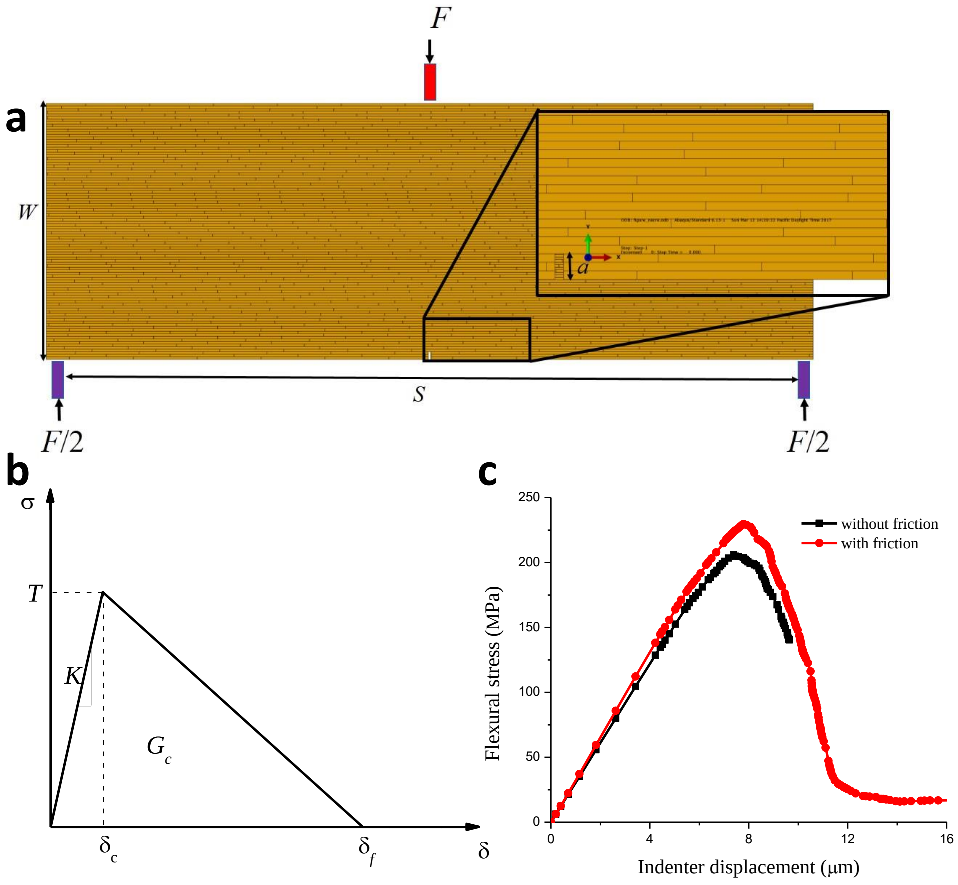

Supplementary Figure 12 | Schematic of the Abaqus simulation model. a, The initial configuration of the 3D brick-and-mortar (BM) structure under three-point bending. **b**, Bilinear traction-separation response of the cohesive element. **c**, Plot of the indenter displacement and flexural stress in three-point bending with and without friction using Abaqus simulation.

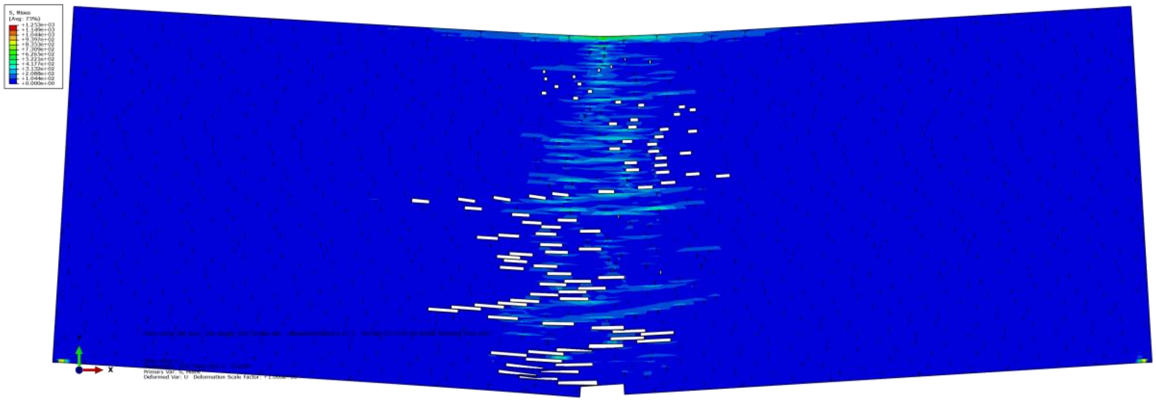

Supplementary Figure 13│Simulated microcrack deflection and crack bridging in a typical 'brick-and-mortar' structure at a larger scale via nonlinear finite element model (FEM) simulation

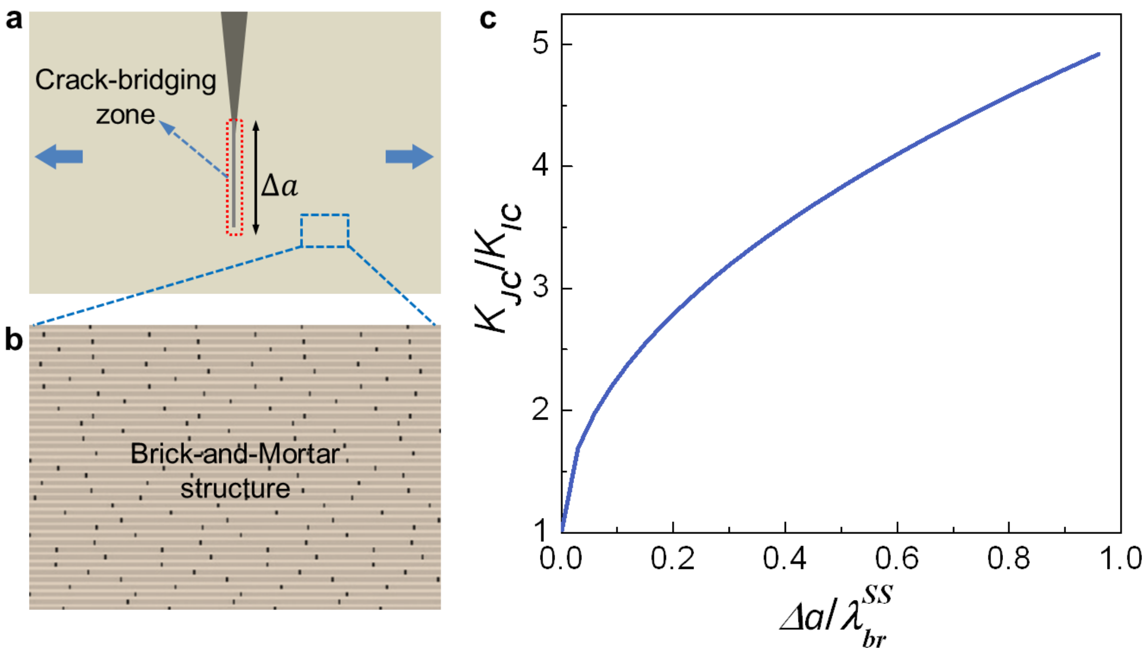

Supplementary Figure 14 | Explanation of the enhanced fracture toughness by crack-bridging. a, The crack growth at the notch produces a crack-bridging zone, whose size is dependent on (**b**) the brick-and-mortar structure. **c**, Before the crack-bridging zone is saturated the fracture toughness monotonically increases.

Supplementary Table 1 │ Size and mechanical properties of brushite platelets for constructing the artificial nacre. The average diameter and thickness of the brushite platelets were determined by calculating them from SEM images, where at least 20 measurements of the brushite platelets were taken. The stiffness and hardness of brushite platelets were measured by nanoindentation.

**Diameter (µm)**	**Thickness (nm)**	**Stiffness (GPa)**	**Hardness (GPa)**
8.4 (s.d. = 2.4)	272.8 (s.d. = 47.7)	55.4 (s.d. = 7.1)	2.4 (s.d. = 0.6)

Supplementary Table 2 │ Comparison of densities of artificial nacres fabricated with different conditions with natural *Cristaria plicata* nacre and other 3D bulk nacre-mimetic materials.

**Samples**	**Density (g cm^{-3})**
Artificial nacre (25 MPa)	1.73 (s.d. = 0.04)
Artificial nacre (50 MPa)	1.81 (s.d. = 0.02)
Artificial nacre (100 MPa)	1.83 (s.d. = 0.02)
Natural *Cristaria plicata* nacre	2.58 (s.d. = 0.07)
Synthetic nacre[10]	2.18
Alumina/PMMA[11]	2.52
Alumina/Copper[11]	3.96
Nacre-like alumina[11]	3.94

Supplementary Table 3 │ Mechanical properties of natural nacre and our bulk artificial nacre fabricated with different conditions.

Platelets Size (μm)	Platelets content (wt.%)	Film thickness (μm)	Applied pressure (MPa)	Glue	Ca^{2+} crosslink-ing	Flexural strength (MPa)	Flexural modulus (GPa)	Flexural Strain (%)
8	50	50	25	CS	Yes	199.3 (s.d. = 11.3)	14.8 (s.d. = 2.2)	2.3 (s.d. = 0.4)
8	50	100	25	CS	Yes	192.7 (s.d. = 16.4)	13.9 (s.d. = 3.1)	2.5 (s.d. = 0.6)
8	50	150	25	CS	Yes	77.9 (s.d. = 17.9)	7.5 (s.d. = 1.7)	2.8 (s.d. = 0.2)
8	50	75	25	CS	Yes	221.3 (s.d. = 9.9)	17.5 (s.d. = 1.3)	1.9 (s.d. = 0.2)
8	50	75	50	CS	Yes	234.9 (s.d. = 19.1)	17.0 (s.d. = 2.7)	2.1 (s.d. = 0.4)
8	50	75	100	CS	Yes	266.6 (s.d. = 24.5)	18.6 (s.d. = 4.6)	2.3 (s.d. = 0.8)
8	40	75	100	CS	Yes	172.7 (s.d. = 23.2)	15.1 (s.d. = 3.4)	2.4 (s.d. = 0.5)
8	60	75	100	CS	Yes	179.0 (s.d. = 13.4)	20.5 (s.d. = 6.0	1.3 (s.d. = 0.5)
8	50	75	100	SA	Yes	149.3 (s.d. = 23.6)	15.1 (s.d. = 5.1)	2.2 (s.d. = 1.0)
8	50	75	100	CS	No	168.1 (s.d. = 11.9)	11.5 (s.d. = 3.0)	4.8 (s.d. = 1.9)

Supplementary Table 4│Comparation of mechanical performance of the designed bulk artificial nacre with the pure SA bulk.

**Mechanical performance**	**Bulk artificial nacre**	**Pure SA bulk**	**Disordered composite**	**Natural *Cristaria plicata* nacre**
Flexural Strength (MPa)	267 (s.d. = 24.5)	155 (s.d. = 6.4)	90.8 (s.d. = 24.1)	171.7 (s.d. = 51.4)
Stiffness (GPa)	18.6 (s.d. = 4.6)	2.4 (s.d. = 0.2)	10.2 (s.d. = 1.5)	48.9 (s.d. = 12.1)
Flexural Strain (%)	2.3 (s.d. = 0.8)	10.9 (s.d. = 2.8)	1.2 (s.d. = 0.2)	0.6 (s.d. = 0.2)
Impact Strength (KJ m^{-2})	7.1 (s.d. = 1.0)	10.5 (s.d. = 2.7)	-	1.4 (s.d. = 1.0)
Vickers Hardness (Hv 1.0) (kg mm^{-2})	64.3 (s.d. = 3.6)	36.0 (s.d. = 4.8)	-	-
Fracture Toughness K_{Ic} (MPa $m^{1/2}$)	1.9 (s.d. = 0.8)	1.9 (s.d. = 0.3)	1.2 (s.d. = 0.41)	2.4 (s.d. = 0.52)
Maximum Toughness K_{Jc} (MPa $m^{1/2}$)	8.7 (s.d. = 1.2)	4.9 (s.d. = 0.6)	3.8 (s.d. = 0.49)	5.9 (s.d. = 0.85)

Supplementary Table 5 | Constituent content in the ultimate bulk artificial nacre calculated from organic elemental analysis results.

Constituents	Mass fraction (wt.%)
CS	5.9 (s.d. = 0.9)
SA	44.5 (s.d. = 3.5)
Brushite platelets	49.6 (s.d. = 2.7)

Supplementary References

1 Glavinch.B & Piggott, M. Steel Disk Reinforced Polycarbonate. *J. Mater. Sci.* **8**, 1373-1382 (1973).

2 Bonderer, L. J., Studart, A. R. & Gauckler, L. J. Bioinspired design and assembly of platelet reinforced polymer films. *Science* **319**, 1069-1073 (2008).

3 Ni, Y., Song, Z. Q., Jiang, H. Y., Yu, S. H. & He, L. H. Optimization design of strong and tough nacreous nanocomposites through tuning characteristic lengths. *J. Mech. Phys. Solids* **81**, 41-57 (2015).

4 Begley, M. R. et al. Micromechanical models to guide the development of synthetic 'brick and mortar' composites. *J. Mech. Phys. Solids* **60**, 1545-1560 (2012).

5 Barthelat, F. Designing nacre-like materials for simultaneous stiffness, strength and toughness: Optimum materials, composition, microstructure and size. *J. Mech. Phys. Solids* **73**, 22-37 (2014).

6 Gao, H. J., Ji, B. H., Jager, I. L., Arzt, E. & Fratzl, P. Materials become insensitive to flaws at nanoscale: Lessons from nature. *P. Natl. Acad. Sci. USA* **100**, 5597-5600 (2003).

7 Shao, Y., Zhao, H. P., Feng, X. Q. & Gao, H. J. Discontinuous crack-bridging model for fracture toughness analysis of nacre. *J. Mech. Phys. Solids* **60**, 1400-1419 (2012).

8 Bui, Q. V. A modified Benzeggagh-Kenane fracture criterion for mixed-mode delamination. *J. Compos. Mat.*, **45**, 389-413 (2011).

9 Zolotoyabkoa, E. Determination of the degree of preferred orientation within the March-Dollase approach. *J. Appl. Crystallogr.*, **42**, 513-518 (2009).

10 Mao, L. B. et al. Synthetic nacre by pre-designed matrix directed mineralization. Science, **354**, 107-110 (2016).

11 Bouville, F. et al. Strong, tough and stiff bioinspired ceramics from brittle constituents. *Nat. Mater.* **13**, 508-514 (2014).
